# Can the Virtual Reality-Based Spatial Memory Test Better Discriminate Mild Cognitive Impairment than Neuropsychological Assessment?

**DOI:** 10.3390/ijerph19169950

**Published:** 2022-08-12

**Authors:** Jin-Hyuck Park

**Affiliations:** Department of Occupational Therapy, College of Medical Science, Soonchunhyang University, Asan 31538, Korea; roophy@naver.com; Tel.: +82-43-530-4773

**Keywords:** cognitive function, mild cognitive impairment, spatial memory, virtual reality

## Abstract

Neuropsychological screening tools for mild cognitive impairment (MCI) have been widely used. However, to date, their sensitivity and specificity still remain unsatisfied. This study aims to investigate whether spatial memory can discriminate MCI better than neuropsychological screening tools. A total of 56 healthy older adults and 36 older adults with MCI participated in this study; they performed a spatial cognitive task based on virtual reality (SCT-VR), the Korean version of the Montreal Cognitive Assessment (MoCA-K), and the Wechsler Adult Intelligence Scale-Revised Block Design Test (WAIS-BDT). The discriminant power was compared between the SCT-VR and the MoCA-K, and the reliability and validity of the SCT-VR were analyzed. The spatial memory, assessed by the SCT-VR, showed better sensitivity and specificity (sensitivity: 0.944; specificity: 0.964) than the MoCA-K (sensitivity: 0.857; specificity: 0.746). The test-retest reliability of the SCT-VR was relatively high (ICCs: 0.982, *p* < 0.001) and the concurrent validity of the SCT-VR with the MoCA-K (r = −0.587, *p* < 0.001) and the WAIS-BDT (r = −0.594, *p* < 0.001) was statistically significant. These findings shed light on the importance of spatial memory as a behavioral marker of MCI. The ecologically validated spatial memory tasks based on VR need to be investigated by neuroscientific studies in the future.

## 1. Introduction

Mild cognitive impairment (MCI) is a clinical pre-stage of dementia. Previous studies indicated that an impairment in episodic memory and spatial cognition, mainly depending on hippocampal function, is one of hallmarks of older adults with MCI even though other cognitive domains have deteriorated [1]. Given that over 10% of patients with MCI experience Alzheimer’s disease (AD) within three years, it is important to discriminate older adults with MCI early before they show obvious clinical signs of AD to reduce enormous social costs [2].

To date, in clinical settings, the Mini-Mental Status Examination (MMSE) and the Montreal Cognitive Assessment (MoCA) have been used to distinguish older adults with MCI from healthy older adults [3]. Specifically, the MMSE has been proven valid and reliable to use for the screening of patients with cognitive impairment within 10 min. However, the sensitivity of the MMSE to MCI is thought to be very low since it can be highly affected by the education levels or age of the subjects. Indeed, a previous study reported that the MMSE is inappropriate to test older adults with MCI [4]. Although the MoCA was developed to complement the MMSE, the relative weight of the items for an episodic memory test in the MoCA is low, resulting in low sensitivity to discriminate MCI, as well as the MMSE [3]. In addition, both the MMSE and the MoCA could be influenced by the proficiency of the tester, as well as by the test environment [5].

Thus, currently, in order to overcome these issues, hippocampal-dependent episodic memory tasks have been used. Several studies suggested that cognitive tasks for testing episodic memory could better discriminate older adults with MCI from normal older adults [6]. Specifically, among the sub-elements of episodic memory, spatial location memory and temporal order memory showed a higher predictive power of MCI than conventional neuropsychological assessments, such as verbal learning tests [6,7]. Compared with these neuropsychological assessments that assess only object memory by asking the subjects to recall what they had to remember, cognitive tasks requiring them to remember sub-elements of episodic memory in spatiotemporal contexts can lead to a higher predictive power [6,7].

Accordingly, spatial memory, depending on hippocampal function, could also be used for screening for MCI. In a previous study, spatial memory has been evaluated by using tasks for remembering locations of objects or pictures presented on a computer screen or a table top to discriminate MCI [8]. However, spatial memory assessed in a two-dimensional array depends on other brain areas rather than hippocampal function, resulting in its low predictive power [9]. Therefore, in order to test the hippocampal-dependent spatial memory, the cognitive tasks need to involve a complex navigational situation in a three-dimensional environment. Recently, with the development of virtual reality (VR), VR has been applied to a variety of cognitive tasks [7,10]. Indeed, a previous study evaluated episodic memory in a spatiotemporal context of older adults with MCI, who traveled within a city in a VR environment based on their city tour experiences [7]. However, there is still a lack of evidence that spatial memory could be used as a factor for discriminating MCI compared to conventional neuropsychological screening tools.

Therefore, the first objective of this study was to determine whether the virtual reality-based spatial memory task has a higher predictive power of MCI than a conventional neuropsychological screening tool. The second objective was to identify the most effective behavioral marker of MCI.

## 2. Materials and Methods

### 2.1. Participants and Procedure

All participants were recruited from senior centers and welfare centers in Seoul, Republic of Korea. The eligibility criteria were as follow: (1) over 65 years old, (2) an absence of neurological or psychiatric disorders, (3) agreement to participate in the present study. All participants were assigned to the healthy control (HC) group or the MCI group.

The HC group consisted of 56 older adults with no memory complaints, and it was confirmed that they were in the normal range by using neuropsychological assessments. The MCI group consisted of 36 elderly persons with amnestic MCI (a-MCI). A-MCI was defined in accordance with the criteria of Petersen et al. (2004) [11] as follows: (1) a subjective memory complaint, (2) a memory decline compared with age- and education level-matched healthy older adults confirmed by neuropsychological assessments (below 1.5 standard deviation), (3) intact global cognitive function confirmed by scores of the Korean version of the Mini-Mental State Examination (MMSE-K), (4) intact activities of daily living (ADL), and (5) not diagnosed with dementia by a physician. For the neuropsychological assessment, the Seoul Neuropsychological Screening Test (SNSB) was used for each participant [12]. All assessments were implemented by a blinded assessor who is familiar with the SNSB.

All participants completed an informed consent form before participating in this study; this study was approved by the institutional review board of Soonchunhyang University. All participants performed the spatial cognitive task based on virtual reality (SCT-VR), the Wechsler Adult Intelligence Scale-Revised Block Design test (WAIS-BDT), and the Korean version of the MoCA (MoCA-K). These outcome measurements were conducted on the same day for each participant, and the order of the measurements was randomized.

### 2.2. Measures

#### 2.2.1. Spatial Cognitive Task Based on Virtual Reality (SCT-VR)

The SCT-VR, programmed in the Unity game engine, was implemented by using a desktop in a quiet room. The participants used a joystick to freely move in the virtual environment. During the SCT-VR, the participants were immersed in an open arena environment with boundary cues, such as an ocean and rocks. No landmarks were provided in the task in order to exclude participants’ compensatory navigation. Before starting the test sessions, the participants underwent two sessions in order to enable them to familiarize themselves with the control of the joystick and the environment until they were accustomed to the task. In the test sessions, the participants started at a location that was randomly selected in the environment, and they were asked to look around. There was a gem at a point in the environment where the participants were instructed to move toward. After the participants reached it, another gem was presented at a location that differed from the previous one, and they were asked again to reach it. Upon reaching the second gem, a message projected onto the virtual environment instructing the participants to walk back to their starting location. When they moved to the location they estimated, they pressed a button on the joystick that confirmed their location and ended the session. The participants performed a total of 10 sessions, and the Euclidean distance between the starting location and the estimated location was recorded [10]. In this study, the Euclidean distance average of 10 sessions as spatial memory was used for an analysis. To evaluate the test-retest reliability, 30 of the participants, who were randomly selected, performed the task again after 2 weeks. 

#### 2.2.2. Wechsler Adult Intelligence Scale-Revised Block Design Test (WAIS-BDT)

The WAIS-BDT includes a task that requires the participants to arrange nine colored blocks in order to replicate ten different patterns within 120 s from a low to a high level. The WAIS-BDT score ranges from 0 to 48, with higher scores indicating better spatial cognition [13].

#### 2.2.3. Korean Version of the Montreal Cognitive Assessment (MoCA-K)

In this study, the MoCA-K was used to evaluate global cognitive function. The MoCA-K was developed and standardized for Korean older adults by translating the original MoCA. The MoCA-K consists of items to evaluate visuospatial/executive function, naming, memory, attention, language, abstraction, and orientation. The total score of the MoCA-K can range from 0 to 30, and the higher scores indicate better global cognitive function, with the cut-off of 23 points [14].

### 2.3. Statistical Analyses

All data in this study were analyzed by using the SPSS statistics (version 25.0, IBM, Armonk, NY, USA). Descriptive statistics were used to identify the demographic characteristics of the participants. The test-retest reliability of the spatial cognitive task was tested by using the intra-class correlation coefficients (ICCs), and the concurrent validity was evaluated using Spearman’s rank correlation. Independent t-tests were used to investigate the differences in the outcome measures between the HC group and the MCI group. A Receiver Operating Characteristic (ROC) analysis was used to confirm the sensitivity and specificity of the outcome measures, and the cut-off scores for MCI were found in accordance with the highest Youden Index.

## 3. Results

### 3.1. Demographic Characteristics

Table 1 indicates participants’ demographic characteristics. As the results show, there were no significant differences in age, sex ratio, education levels, and MMSE-K scores between both groups.

### 3.2. Group Differences in Outcome Measurements

There were significant differences in all the outcome measurements (shown in Table 2). The HC group showed higher performance in the SCT-VR(_t_(90) = 15.157, *p* < 0.001), the WAIS-BDT(_t_(90) = 10.441, *p* < 0.001), and the MoCA-K(_t_(90) = 10.226, *p* < 0.001) than the MCI group, suggesting that the HC group outperformed in both spatial cognition and global cognition.

### 3.3. Reliability and Validity

Test-retest reliability data from 30 participants at a two-week interval were collected. The mean change in the SCT-VR distance error from the first to the second test was from 65.81 to 66.28. The SCT-VR distance error (ICCs = 0.982, *p* < 0.001) showed a high test-retest reliability (Table 3).

The correlations with the MoCA-K were analyzed to identify the concurrent validity of the SCT-VR. As the results show, the SCT-VR distance errors were found to be highly and negatively correlated with the MoCA-K scores (r = −0.587, *p* < 0.001) and the WAIS-BDT scores (r = −0.594, *p* < 0.001), which suggests that the SVT-VR is concurrently validated (shown in Table 4).

### 3.4. Sensitivity, Specificity, and Cut-Off Score

**Figure 1:**
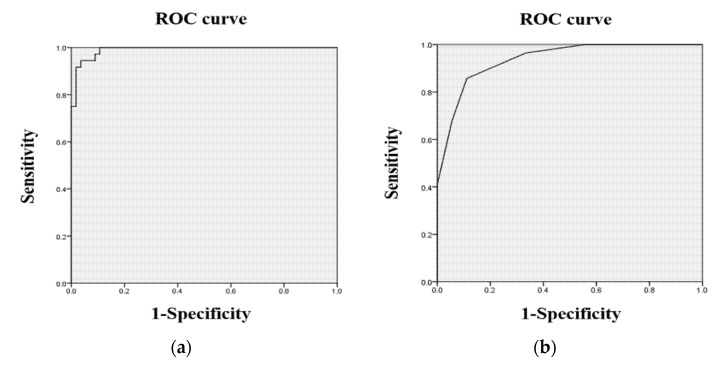
(**a**) ROC curve of the SCT-VR scores; (**b**) ROC curve of the MoCA-K scores.

ROC curves were drawn to determine the discriminator validity of the SCT-VR and the MoCA-K (see Figure 1). The areas under the curve (AUC) for the MCI group by the SCT-VR and the MoCA-K were 0.991 and 0.938, respectively. These findings showed that the SCT-VR could discriminate MCI well compared to the MoCA-K.

The sensitivity, specificity, positive predictive value (PPV), and negative predictive value (NPV) of the SCT-VR and the MoCA-K by different cut-off scores were indicated in Table 5 and Table 6. As the results show, the optimal cut-off score for the SCT-VR scores was identified to be 70.77/71.56. At this score, the sensitivity and specificity of the SCT-VR scores were 94.4% and 96.4%, respectively. Meanwhile, at the 24.50/25.50 cut-off scores, the sensitivity and specificity of the MoCA-K scores were 85.7% and 88.9%, respectively.

For the SCT-VR scores, the PPV and NPV were 94.44% and 96.42%, respectively. On the other hand, for the MoCA-K, the PPV and NPV were 78.94% and 92.30%, respectively (see Table 7).

## 4. Discussion

The current study aimed to investigate whether hippocampal-dependent spatial memory can better discriminate a-MCI from healthy aging than conventional neuropsychological assessments. The present findings reported that spatial memory, as assessed distance errors in the SCT-VR, showed a higher sensitivity and specificity than that of the MoCA-K. In addition, spatial memory was significantly correlated with the MoCA-K, suggesting that spatial memory is concurrently valid as a screening tool for a-MCI. These findings shed new light on the promise of spatial memory in a 3D environment to distinguish a-MCI from normal aging with a higher discriminant power than the MoCA-K, which is supported by a previous study [7].

Numerous studies have reported that hippocampal atrophy is a typical sign of a-MCI, causing spatial memory impairment [15,16], which is consistent with the current study revealing a significant spatial memory decline in the a-MCI group compared to the HC group. Deficits in spatial memory have been widely used as a useful early index of MCI, supporting the present findings [6,7]. Indeed, a previous study using VR to characterize episodic memory profiles in a-MCI reported that spatial memory is particularly considerable for discriminating a-MCI [7]. Taken together, these results suggest that the items to test spatial memory need to be included in the screening tools to efficiently distinguish a-MCI from healthy aging. On the other hand, although the MoCA-K is widely used for screening for MCI, it has a relatively low discriminant power [3,17]. The key point underlying its inferiority is based on the nature of the MoCA-K. The MoCA-K includes visuospatial/executive function items with 5 out of 30 points assigned, indicating that the test items related to spatial memory are equally distributed rather than weighted. This characteristic might deteriorate the discriminant power of the MoCA-K compared to spatial memory, which is consistent with previous studies [3,17].

Accordingly, several studies have tried to investigate the clinical usefulness of spatial memory as an indicator of MCI [6,7,15]. Some previous studies assessed spatial memory using two-dimensional spatial tasks. These two-dimensional tasks mainly require the use of an egocentric representation with a body-centered viewpoint to remember spatial locations [10,18]. However, numerous prior studies have reported that the hippocampus is more active when addressing spatial tasks with an allocentric representation rather than with an egocentric representation [18,19]. Indeed, the egocentric representation is thought to be more correlated with posterior parietal function [19], which is supported by the fact that individuals with MCI retained an ability to use an egocentric representation with preserved posterior parietal function. These results advocated that older adults with a-MCI in this study were assessed by three-dimensional spatial tasks with an allocentric representation. Previous studies reported that allocentric spatial memory, which is assessed by three-dimensional spatial tasks in VR environments, is the most powerful predictive indicator, supporting the present findings [7,15].

On the other hand, a VR-based spatial task has the advantage in terms of ecological validity. Conventional paper-based spatial tasks or computerized spatial tasks contrast with the requirements of daily life, and the conditions of these tasks are far from ecological [7]. Thus, numerous studies indicated that neuropsychological assessments need to deal with the demands of daily life. As part of this effort, recent studies take advantage of VR to provide conditions that resemble daily life [20]. In prior studies, people with MCI showed a close relationship between performance in VR and the real world, which implies that the findings of this study have ecologically valid implications [21,22].

Even though, this study has clinical implications, there are limitations. First, although this study tried to ecologically validate the spatial cognitive task by using a VR environment, the real world requires participants to actually move, leading to the limitation that actual physical movement could affect spatial memory [15]. Second, activity in the hippocampus was directly observed by neuroimaging methods, such as functional magnetic resonance imaging (fMRI). Thus, it is not clear whether the performance on the SCT-VR relied on the hippocampus or other compensatory mechanisms (e.g., visual scene recognition). Nevertheless, there is a numerous amount of evidence that the hippocampus is activated during spatial memory tasks with an allocentric strategy, supporting that the SCT-VR depends on hippocampal function. Third, recent evidence indicates that the entorhinal cortex is one of the earliest areas to atrophy in MCI [6]. Since temporal memory mainly depends on the entorhinal cortex, spatial memory tasks combined with temporal aspects need to be investigated to find out the more efficient behavioral markers of MCI. Therefore, future studies should investigate the neural correlates of this task with neuroimaging data and compare spatial memory with temporal memory.

## 5. Conclusions

To conclude, the present study showed that spatial memory might be a clinically useful tool in distinguishing people with MCI from older adults with healthy aging. Spatial memory in a VR environment could be implemented in a safe manner in one’s daily life instead of performing neuropsychological assessments that take a lot of time and are not very sensitive to MCI. The findings of this study will help guide the design of VR-based spatial memory tasks targeting the hippocampal function in the near future.

## Figures and Tables

**Table 1:** General characteristics of the participants (*n* = 92).

Characteristics		MCI (*n* = 36)	HC (*n* = 56)	χ^2^/t
Age, years (SD)		75.33 (6.18)	73.02 (6.47)	1.704
Sex, N (%)	Male	14 (38.9)	25 (44.6)	0.297
	Female	22 (61.1)	31 (55.4)	
Education, years (SD)		5.50 (4.45)	6.25 (4.35)	0.800
MMSE-K scores (SD)		26.25 (1.25)	26.27 (1.44)	0.061

MMSE-K, Mini-Mental Status Examination; SD, Standard deviation.

**Table 2:** Group differences in outcome measurements (*n* = 92).

Outcome Measurements	MCI (*n* = 36)	HC (*n* = 56)	t
SCT-VR, mm (SD)	84.94 (8.99)	55.04 (9.38)	15.157 ***
WAIS-BDT, scores (SD)	19.50 (2.96)	25.95 (2.84)	10.441 ***
MoCA-K, score (SD)	22.50 (1.92)	26.30 (1.61)	10.226 ***

*** *p* < 0.001.

**Table 3:** Test-retest reliability of the SCT-VR (*n* = 30).

Variable	ICCs	95% Confidence Interval
Distance error, mm	0.982 ***	0.962–0.991

*** *p* < 0.001.

**Table 4:** Concurrent validity of the SCT-VR (*n* = 92).

Variable	MoCA-K Score	WAIS-BDT Score
Distance error, mm	−0.587 ***	−0.594 ***

*** *p* < 0.001

**Table 5:** Sensitivity and specificity of the SCT-VR scores for the detection of MCI (*n* = 92).

Cut-Off	Sensitivity	Specificity	Youden Index
69.81/70.35	0.944	0.929	0.873
70.35/70.77	0.944	0.946	0.891
70.77/71.56	0.944	0.964	0.909
71.56/73.15	0.917	0.964	0.881
73.15/74.19	0.917	0.982	0.899
74.19/74.64	0.889	0.982	0.871

**Table 6:** Sensitivity and specificity of the MoCA-K scores for the detection of MCI (*n* = 92).

Cut-Off	Sensitivity	Specificity	Youden Index
22.50/23.50	1.000	0.444	0.444
23.50/24.50	0.964	0.667	0.631
24.50/25.50	0.857	0.889	0.746
25.50/26.50	0.679	0.944	0.623
26.50/27.50	0.411	1.000	0.411

**Table 7:** Comparison between PPV and NPV based on the cut-off between the SCT-VR and the MoCA-K (*n* = 92).

Cut-Off	Classification		Predictability
	MCI Group	HC Group	
SCT-VR distance error	34	2	PPV: 94.44%
	2	54	NPV: 96.42%
MoCA-K score	30	8	PPV: 78.94%
	4	48	NPV: 92.30%

## Data Availability

The data presented in this study are available on request from the corresponding author. The data are not publicly available because they are part of an ongoing project.
